# Influence of mixed ventilation on particulate-gas diffusion and distribution of diesel engine exhaust in fully mechanized excavation face

**DOI:** 10.1038/s41598-023-27812-z

**Published:** 2023-01-28

**Authors:** Gang Zhou, Yang Yang, Jinjie Duan, Bin Jing, Shuzheng Song, Biao Sun

**Affiliations:** 1 College of Safety and Environmental Engineering, Shandong University of Science and Technology, Qingdao, 266590 China; 2 State Key Laboratory of Mining Disaster Prevention and Control Co-founded by Shandong Province and the Ministry of Science and Technology, Shandong University of Science and Technology, Qingdao, 266590 China; 3 Nanjing Design and Research Institute, China Coal Technology and Engineering Group, Nanjing, 210031 China

**Keywords:** Health occupations, Environmental sciences, Environmental chemistry, Environmental impact

## Abstract

Tail gas emitted by underground trackless rubber wheel cars poses a serious threat to the health and safety of underground workers. To effectively reduce the tail gas concentration of a comprehensive excavation face, this study adopted a numerical simulation method to investigate the influence of air suction volume Q and distance L between trackless rubber wheel cars and headfaces on the diffusion law of diesel particulate matter, CO, and NO_x_ under long suction and short pressure ventilation. The results showed that under the condition of L = 20 m, the trackless rubber wheel car is closer to the suction air duct. At this point, when Q = 600 m^3^/min, the tail gas control effect in the roadway is optimum. In addition, under the condition of L = 40 m, the trackless rubber wheel car is in the middle of the roadway. At this point, when Q = 300 m^3^/min, the tail gas control effect in the roadway is optimum. When L = 60 m and Q = 200 m^3^/min, the ventilation mode in the roadway is mainly pressure-in ventilation. The high-volume-fraction NO_x_ region and the medium-volume-fraction NO_x_ region under this air volume are small.

## Introduction

Coal is crucial for China's industrial development^1–3^. Every year, China's coal consumption accounts for more than 50% of the country’s total energy consumption^4–6^. With the improvement in mine mechanization levels, the demand of mine enterprises for underground auxiliary transportation is increasing^7–9^. Trackless rubber wheel cars are widely used in large mines because of their flexibility and convenience. The use of a trackless rubber wheel car in a comprehensive excavation face greatly improves the efficiency of underground material transportation and reduces labor intensity for miners^10–12^. However, owing to narrow space of the comprehensive excavation working face, the tail gas released by the trolley accumulates in the working place and causes serious harm to the miners. The tail gas released by the trackless rubber wheel vehicle mainly comprises diesel particulate matter (DPM), CO, and NO_x_. Several toxic chemicals are present on the surface of DPM, which can cause serious damage to the human respiratory system^13,14^. When NO_x_ enters the alveoli, nitrite and nitric acid are formed, which have a severe stimulatory effect on the lung tissue. After inhalation, CO can easily bind with hemoglobin in the blood, resulting in hypoxia, headache, dizziness, vomiting, and other symptoms. Therefore, it makes sense to study the influence of underground ventilation on the discharge of toxic substances during mine safety production^15,16^.

Commonly used diesel exhaust purification technology is mainly divided into two categories: internal and external purification. Ji et al.^17^ added a small amount of metal additive Ce to diesel oil and observed that with an increase in Ce content, HC, CO, and particulate matter in diesel engine exhaust decreased significantly; however, its NO_x_ content increased. Lou et al. established a simulation model of a diesel engine particle catcher (DPF) based on GT-Power and analyzed the trapping process of DPM by the DPF^18^. Although internal and external purification can control the emission of DPM, they still have the disadvantages of producing other toxic and harmful substances, and require frequent replacement of the disposable filters. For the humid and dusty underground environment, ventilation is adopted in mines to dilute and disperse the exhaust gas. Kurnia et al. proposed innovative ventilation techniques using computational fluid dynamics (CFD) methods to evaluate downhole airflow, oxygen, and noxious gas dispersion^19^. The results showed that the proposed ventilation design can deal with the emissions of harmful gases efficiently. Fava et al. proposed a hybrid method for studying the concentration distribution of DPM in underground mines using ventilation network solvers and CFD. The calculation efficiency of the ventilation model was high and accurate, and consequently, detailed results were obtained^20^. Thiruvengadam et al. used the material transport model and discrete phase model in ANSYS FLUENT to conduct numerical simulations of DPM emitted by underground forklift trucks^21^. The results showed that the concentration of DPM simulated by the discrete phase model is close to the actual situation. Xu et al.^22^ studied the influence of the diffusion law of diesel exhaust particles in the roadway through numerical simulation software. Liu et al.^23^ used numerical simulations to study the influence of the diffusion process of wind speed on underground exhaust particles. The results showed that a wind speed of 1.8 m/s can help in alleviating the phenomenon of tail gas particle aggregation. Chang et al. studied the diffusion state of DPM in two underground scenarios by using CFD and verified the simulation results through field measurements^24^. Liu et al. used a method of combining numerical simulation with field measurements to study the distribution state of DPM in the roadway and the dilution effect of air volume on DPM when the trackless rubber wheel vehicle is idled under different underground conditions for 60 s^25^.

The above-mentioned domestic and foreign scholars have conducted a lot of research on the diffusion law of DPM in underground mines, which has provided valuable experience to solve the problem of DPM overload in underground. However, they only considered the harmful factor of DPM, and did not conduct a comprehensive study on the distribution and diffusion law of gas–solid two-phase flow composed of DPM, CO and NO_X_ in the tunnel, meanwhile, the study on the diffusion effect of different ventilation methods on the exhaust pollutants emitted from fuel-powered equipment is still shallow.

Therefore, in this paper, based on Discrete Phase Models and component transport models, we conduct a comprehensive investigation on the dispersion law of diesel exhaust consisting of DPM, CO and NO_X_, consider the dispersion transport effects between different components of exhaust pollutants, and integrate the research theory of gas–solid two-phase flow to analyze the dispersion of exhaust pollutants emitted from trackless rubber wheel vehicles. Meanwhile, the effect of the ventilation system on the distribution of exhaust pollutants emitted from the trackless rubber wheel car was taken into consideration, and the influence of the suction volume Q of the extracted blower and the distance L of the trackless rubber wheel car from the head of the long extraction and short pressure ventilation system on the diffusion law of the locomotive exhaust was studied.

## Mathematical model construction

### Mathematical model of underground airflow movement

The diffusion of dust in driving face and tail gas of trolley is based on the movement of air currents. Therefore, the accuracy of airflow movement law in driving face directly affects the diffusion law of dust and tail gas of trackless rubber wheel vehicle. The flow state of air in coal mine is generally considered to be turbulence^26,27^. At present, Reynolds time-average equation is usually used for turbulence simulation in engineering. The basic idea is to express the transient pulsation in the time-averaged equation through the k-ε two-equation model.

The equation for conservation of mass, or continuity equation, can be written as follows^28,29^:

1 $$\frac{\partial \rho }{{\partial t}} + \nabla \cdot \left( {\rho \vec{\nu }} \right) = S_{m}$$

The equation for the turbulence kinetic energy can be written as follows^30,31^:

2 $$\frac{{\partial \left( {\rho k} \right)}}{\partial t} + \frac{{\partial \left( {\rho ku_{i} } \right)}}{{\partial x_{i} }} = \frac{\partial }{{\partial x_{j} }}\left[ {\left[ {\mu + \frac{{\mu_{t} }}{{\sigma_{\kappa } }}} \right]\frac{\partial k}{{\partial x_{j} }}} \right] + G_{k} + G_{b} - \rho \varepsilon - Y_{M} + S_{k}$$

The equation for the turbulent dissipation rate can be written as follows^32^:

3 $$\frac{{\partial \left( {\rho \varepsilon } \right)}}{\partial t} + \frac{{\partial \left( {\rho \varepsilon u_{i} } \right)}}{{\partial x_{i} }} = \frac{\partial }{{\partial x_{j} }}\left[ {\left[ {\mu + \frac{{\mu_{t} }}{{\sigma_{\varepsilon } }}} \right]\frac{\partial \varepsilon }{{\partial x_{j} }}} \right] + \frac{{C_{1\varepsilon } \varepsilon }}{k}\left( {G_{k} + G_{3\varepsilon } G_{b} } \right) - C_{2\varepsilon } \rho \frac{{\varepsilon^{2} }}{k} + S_{\varepsilon }$$

### Mathematical model of component transport

The diffusion process of diesel exhaust can be regarded as a multi-component transportation problem without chemical reaction. This paper only considers no and CO in tail gas, so the conservation equation of tail gas and air is as follows^33^:

4 $$\frac{\partial }{\partial t}\left( {\rho Y_{i} } \right) + \nabla \cdot \left( {\rho \vec{v}Y_{i} } \right) = - \nabla \cdot \vec{J}_{i} + R_{i} + S_{i}$$

In turbulent flow, the diffusion flux is described by the following equation:

5 $$\vec{J}_{i} = - \left( {\rho D_{i,m} + \frac{{\mu_{t} }}{{Sc_{t} }}} \right)\nabla Y_{i} - D_{T,i} \frac{\nabla T}{T}$$

where ${\text{Sc}}_{t}$ is the turbulent Schmidt number. The default value of ${\text{Sc}}_{t}$ is 0.7.

The equation for the change of harmful gas with time is:

6 $$\frac{\partial }{\partial t}\left( {\rho Y_{m} } \right) + \frac{\partial }{{\partial x_{j} }}\left( {\rho v_{j} Y_{m} } \right) = \frac{\partial }{{\partial x_{j} }}\left( {\frac{{\mu_{c} }}{{\rho_{Y} }}\frac{{\partial Y_{m} }}{{\partial x_{j} }}} \right)$$

where $Y_{m}$ is the mass fraction of the harmful gas.

### Mathematical model of tail gas particle diffusion

Because of the small volume fraction of tail gas particles in the whole flow field, tail gas particles are treated as discrete medium in the fluid, and the discrete phase model is used to describe the trajectory of tail gas particles^34–36^. The specific mathematical equation is as follows:

7 $$m_{p} \frac{{d\vec{u}_{p} }}{dt} = m_{p} \frac{{\vec{u} - \vec{u}_{p} }}{{\tau_{r} }} + m_{p} \frac{{\vec{g}\left( {\rho_{p} - \rho } \right)}}{{\rho_{p} }} + \vec{F}$$

$\tau_{r}$ is calculated by the following equation

8 $$\tau_{r} = \frac{{\rho_{p} d_{p}^{2} }}{18\mu }\frac{24}{{C_{d} Re}}$$

$Re$ is the relative Reynolds number, which is defined as

9 $${\text{Re}} \equiv \frac{{\rho d_{p} \left| {\vec{u}_{p} - \vec{u}} \right|}}{\mu }$$

For the Realizable $k$-$\varepsilon$ model,when the RSM is used, nonisotropy of the stresses is included in the derivation of the velocity fluctuations:

10 $$u^{\prime } = \zeta \sqrt {\overline{{u^{\prime 2} }} }$$

11 $$v^{\prime } = \zeta \sqrt {\overline{{v^{\prime 2} }} }$$

12 $$w^{\prime } = \zeta \sqrt {\overline{{w^{\prime 2} }} }$$

## Geometric model construction and meshing

### Geometric model construction

**Figure 1:**
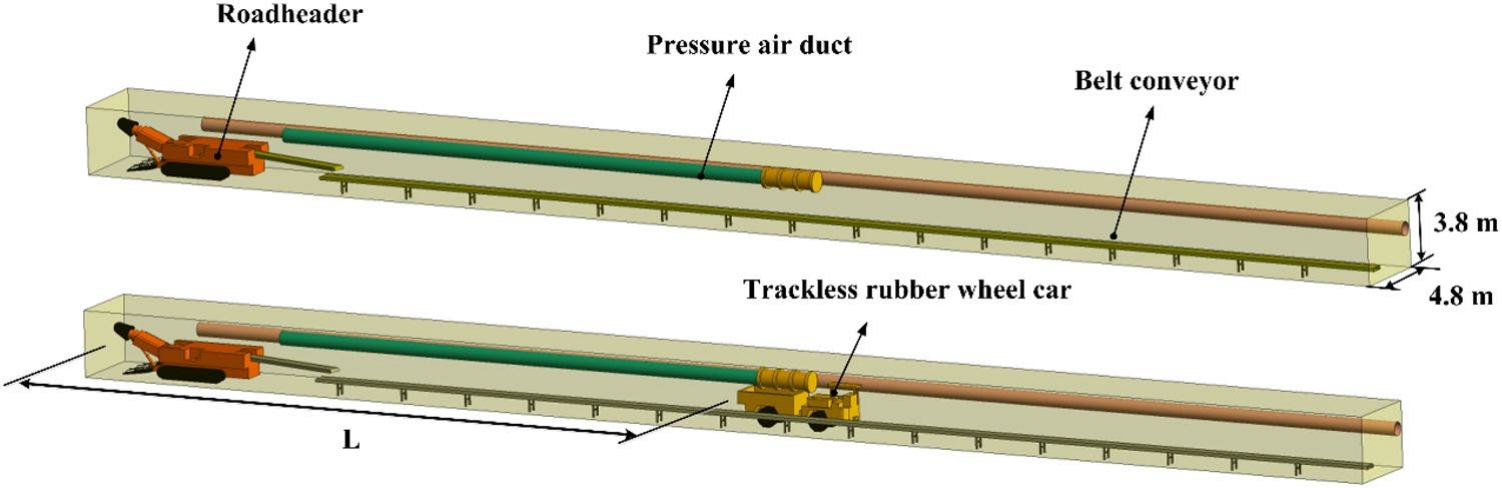
Geometric model of the roadway.

SolidWorks was used to model 15113 return air roadway heading face of Pingshu Company of Yangmei Group. As shown in Fig. 1, the geometric model of the press-in ventilation system consists of five parts: roadways, roadheaders, press-in air ducts, transport belts, and trackless rubber wheel cars. Based on the geometric model of the pressurized ventilation system, a wet dust removal fan was added. The roadway was 80.00 m long, 4.8 m wide, and 3.8 m high; EBZ-200H excavation length was 8.30 m, width was 2.30 m, height was 2.90 m, and was 1.00 m from the side-wall; the suction air duct was 75 m long, 0.8 m in diameter, 1.9 m from the shaft to the roadway floor, and 5 m from the air duct outlet to the head; the pressure-out air duct was 30 m long, 0.8 m in diameter, 3.4 m from the axis to the floor of the roadway, and the distance between the tail of the trackless rubber wheel car and its head was L.

### Grid independence verification

**Figure 2:**
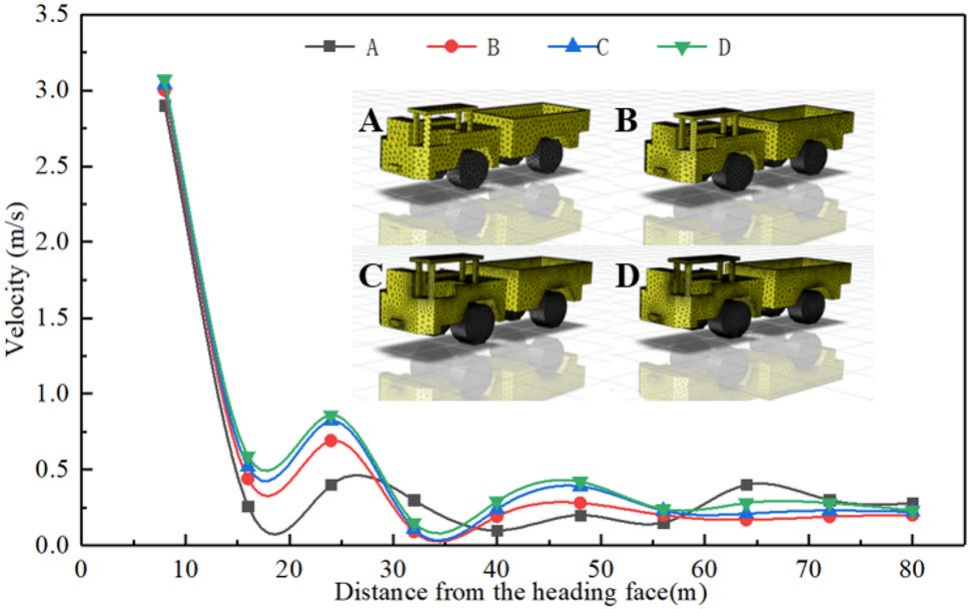
Grid independence verification.

The quality of meshing will affect the accuracy of numerical simulation. Since the research content of this paper is that the trackless rubber wheel vehicle emits tail gas in the static state, the tetrahedral mesh was used to divide the tunnel model as a whole, and then the mesh was locally refined by changing the digital size of “Capture Proximity”. Finally, four groups of grids were obtained, with the number of grids being 750,000, 1,500,000, 3200,000 and 6,000,000 respectively. The mesh inspection tool was used to check the quality of the four groups of meshes, and the inspection results show that the quality of meshes is within the reasonable range of meshes. Fluent was used to simulate the airflow movement in the tunnel under different grids, and the airflow velocity of 10 isotherm points between points (8, 0.8, 1) and points (80, 0.8, 1) was derived through CFD-POST. Finally, independence test was conducted for the four grids, as shown in Fig. 2. It can be seen from the figure that the variation trend of air flow velocity simulated by the four grids is roughly the same, but the results obtained by grids C and D are relatively close, while the results obtained by grids A and B have large deviations. Therefore, considering the simulation accuracy and calculation cost comprehensively, grid C is selected for simulation calculation.

### Parameter setting

AEROTRAK™9306 hand-held laser particle counter and a specific length detector are used to determine the concentrations of particulate, CO, and NO_x_ in the trackless rubber truck exhaust. The mass flow rate of particulate is calculated based on the emission rate and cross-sectional area of the exhaust outlet. Finally, this is used as the parameter of numerical simulation. The specific parameter Settings are shown in Table 1.

**Table 1:** Parameter setting.

Name	Type	Parameter settings
Inlet	Velocit inlet	13.3 m s^−1^
Outlet	Pressure outlet	–
DPM	Distribution mode	Rosin–Rammler
	Mass flow rate (kg s^−1^)	1.3 × 10^−6^
	Min Diameter (m)	1 × 10^−8^
	Max Diameter (m)	1 × 10^−6^
	Mean Diameter (m)	7.87 × 10^−8^
CO source parameters	Species mass fraction (%)	0.001
	Density (kg m^−3^)	1.25
NO_x_ source parameters	Species mass fraction (%)	0.0008
	Density (kg m^−3^)	1.34

## Simulation results and analysis

### Rules of airflow migration under long pumping and short pressure ventilation

**Figure 3:**
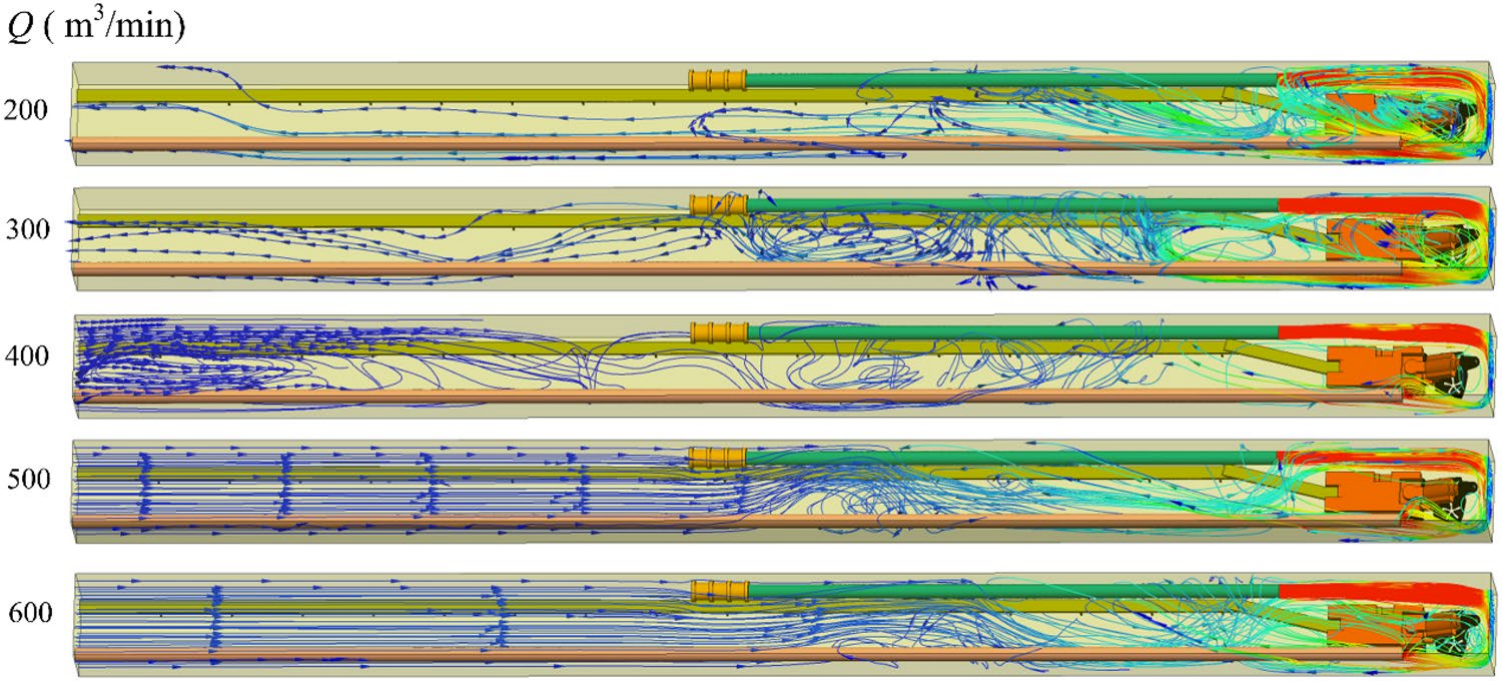
Downdraught flow diagram of air volume Q of different suction ducts.

Figure 3 shows the migration rule of airflow under long and short pressure ventilation. The air volume of the injection-type air duct was fixed at 400 m^3^/min, and the air volume of the extraction-type air duct was increased in a gradient form from 200 to 600 m^3^/min.

1. When the ventilation mode in the roadway was dominated by the pressurized ventilation (when the air volume of the pressurized air duct is greater than that of the extractive air duct), the flow field in the roadway was divided into three areas: “triangular eddy field” near the headface, "turbulent field" in the middle of the roadway, and “advection field” at the tail of the roadway. Within the range of approximately 0–12 m from the headface, the momentum of the high-speed jet ejected from the pressurized air duct suddenly decreased after colliding with the headface and formed a reverse wind flow with a velocity of approximately 5 m/s. Part of the reverse airflow was discharged from the roadway through the suction duct, and the other part of the reverse airflow continued to move toward the exit of the roadway. Owing to high velocity and low pressure, the high-speed jet field generated by the inputting air duct moved near the outlet of the inputting air duct and formed a “triangular vortex field” around the roadheader. Moreover, owing to the continuous negative pressure of the extractor air duct, the velocity of the reverse airflow that continued to move toward the outlet of the roadway gradually decreased from 4 m/s to 0, and the reverse airflow began to move toward the outlet of the extractor air duct. Therefore, a “turbulent flow field” was formed within the range of approximately 12–45 m from the headface. In the “turbulent flow field,” there was a reverse flow toward the exit of the tunnel and a flow toward the exit of the extractor duct. In the range of approximately 45–80 m from the headface, the reverse airflow began to flow smoothly to the exit of the roadway and formed an “advection field” in this area, and the velocity of the airflow in the “advection field” was maintained at approximately 0.2–0.7 m/s.
2. When the air volume of the pressurized air duct was the same as that of the extractor air duct, the flow field in the roadway was mainly categorized into “J-shaped flow field” near the headface and "turbulent flow field" in the middle and back of the roadway. Within the range of approximately 0–12 m from the headface, owing to the increase in the air volume of the extractor, most of the reverse airflow flowed out of the roadway through the extractor, resulting in a loss of the reverse air volume. A small part of the reverse airflow moved toward the back end of the roadway at a speed of 2.5 m/s. Under the negative pressure action of the suction air duct and the negative pressure flow field generated by the high-speed jet, the energy gradually decreased and resulted in a change of direction. In the range of approximately 19–80 m from the headface, the air volume entering the roadway and flowing out of the roadway at the front end was the same. Therefore, there was no large pressure difference between the front end of the roadway and the exit of the roadway, resulting in a small flow field energy in this area and a disorderly flow of air.
3. When the ventilation mode in the roadway was dominated by extraction ventilation (when the air volume of the pressurized air duct was less than that of the extraction air duct), the flow field in the roadway was mainly divided into three areas: “J-shaped flow field” near the headface, "turbulent flow field" in the middle of the roadway, and "backflow field" at the tail of the roadway. Compared with the ventilation mode dominated by pressure-in ventilation, the pressure at the front end of the roadway was less than that at the back end of the roadway owing to the increase in the air volume of the extraction duct; therefore, the air flowed with a velocity of approximately 0.2 m/s from the exit of the roadway to the roadway. At a distance of 36 m from the head, the suction effect of the suction duct on the backflow airflow was enhanced, resulting in an increase of the velocity of the backflow airflow from 0.2 to 1 m/s.

### CO gas migration rule under long pumping and short pressure ventilation

**Figure 4:**
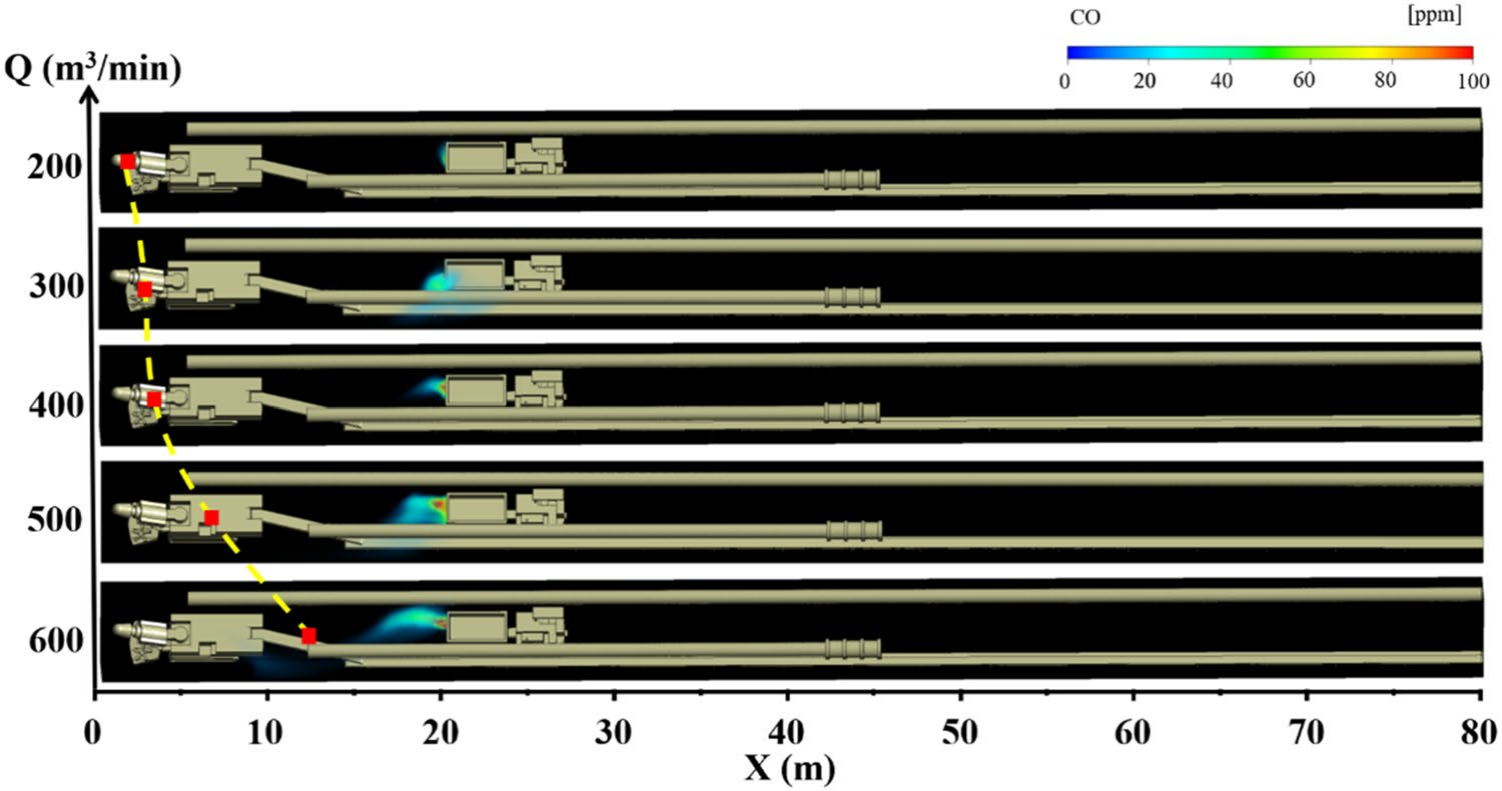
CO gas distribution under different air volumes Q when L = 20 m.

**Figure 5:**
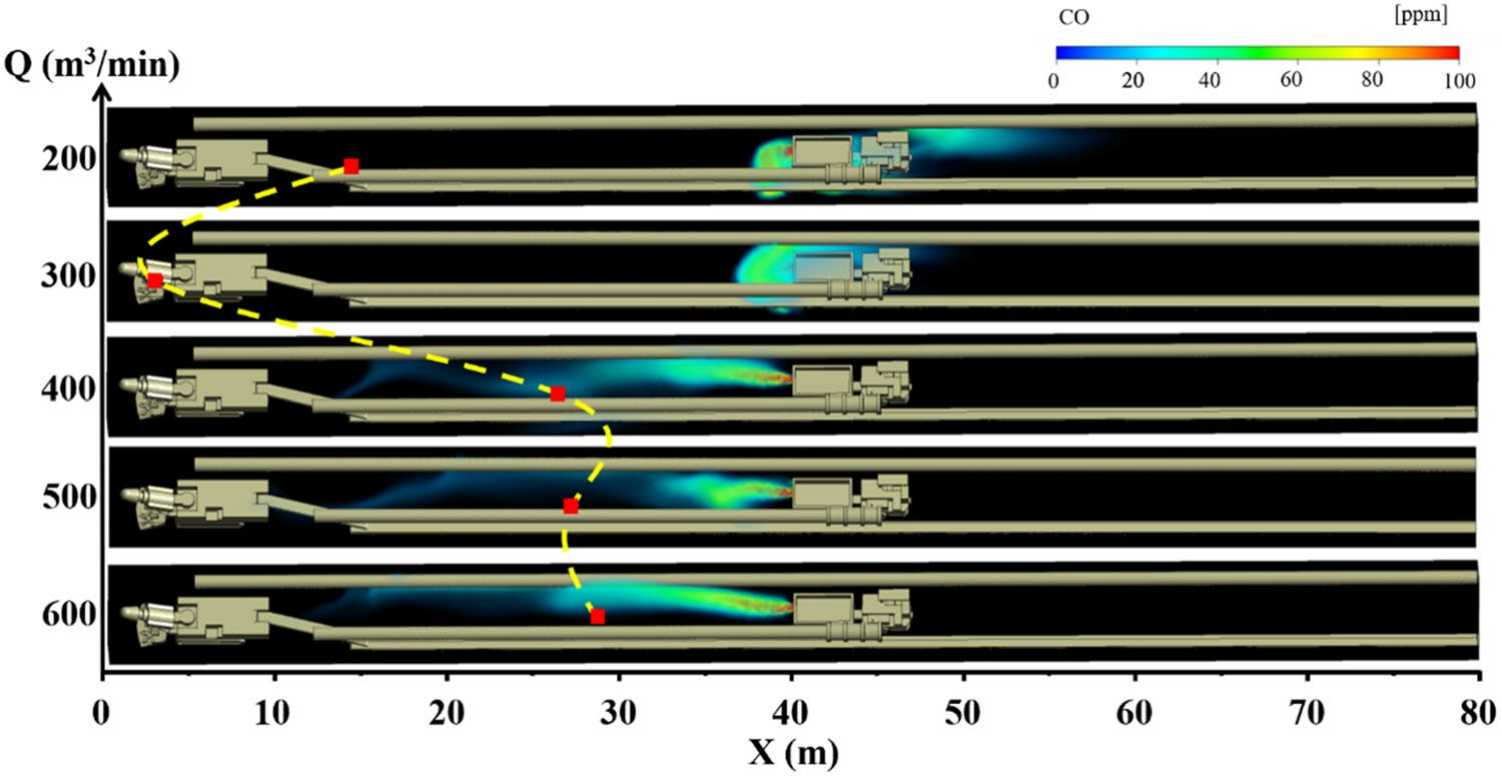
CO gas distribution under different air volumes Q when L = 40 m.

**Figure 6:**
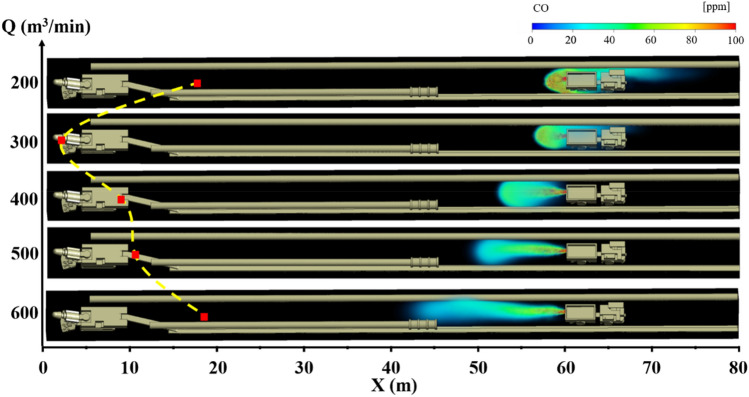
CO gas distribution under different air volumes Q when L = 60 m.

Under the condition of long suction and short pressure ventilation, the distribution of CO gas under different air volumes Q of the suction ducts and the distance L between the trackless rubber wheel car and headface is shown in Figs. 4, 5 and 6. The different colors in the figures indicate the mass fractions of CO gas in different regions. The specific analysis is as follows:

1. When the distance L between the trackless rubber wheel car and the headface was constant, the distribution of CO in the roadway exhibited a certain regularity with the increase in the air volume Q of the extractive air duct. When L = 40 m and Q = 200 m^3^/min, the pressure at the front end of the roadway was greater than that at the exit of the roadway, and the airflow in the roadway migrated from the head to the exit of the roadway. Therefore, CO diffused in the direction of the exit of the roadway. At this time, the diffusion distance of CO was 19 m, and the mass fraction was between 20 and 40 ppm. When L = 40 m and Q = 400 m^3^/min, the trackless rubber wheel vehicle was in the “turbulent flow field,” and the wind speed was 0.45 m/s, the energy was low, and the CO emission from the trackless rubber wheel car was affected negligibly. At this time, CO gas diffused toward the headface at a certain initial velocity, and the diffusion distance was 28 m. When L = 40 m and Q = 600 m^3^/min, CO gas diffused toward the front face under the influence of the negative pressure “J-shaped flow field” at the front of the roadway. When CO diffused 10 m away from the headface, CO gas was involved in the negative pressure flow field formed by the high-speed jet. At this point, the mass fraction of CO was instantly diluted from 20 to 0.1 ppm, and the diffusion distance was 32 m.
2. When the air volume Q of the suction duct was constant, the position of the trackless rubber wheel car in the roadway had little influence on the overall airflow field of the roadway. Therefore, the diffusion state of CO gas was significantly affected by the flow field corresponding to the location of the trackless rubber wheel car. Taking Q = 600 m^3^/min as an example, when L = 20 m, the trackless rubber wheel vehicle was in the “turbulent flow field” and was affected by the suction air duct at the front end of the roadway. CO gas diffused to the front end of the roadway and was discharged through the suction air duct. Therefore, the mass fraction of CO gas at the front of the roadway was very small, at only 0.1 ppm. When L = 40 m, the trolley was in the backflow field. The CO gas discharged by the trolley diffused to the front end of the roadway with a backflow airflow of 1 m/s, and the diffusion distance was 28 m. When L = 60 m, the trolley remained in the backflow field. However, compared with L = 40 m, the distance was far from the headface, and the negative pressure effect of the extractive air duct was affected negligibly by the location, resulting in a backflow airflow velocity of only 0.2 m/s. Therefore, when L = 60 m, the diffusion distance of CO gas to the front end of the roadway decreased, but the mass fraction was high. The diffusion distance and mass fraction of CO gas were 20 m and 40 ppm, respectively.
3. In summary, when L = 20 m, the CO gas control effect was optimum for all air volumes. This is because the location was close to the outlet of the suction air duct. Therefore, the larger the suction volume of the extractor air duct, the faster the CO gas diffuses to the front end of the roadway and is easily discharged from the driving face by the extractor air duct. At this point, the mathematical relationship between the diffusion distance C_20_ of the CO gas and air volume Q is $C_{20} = \;\left( {2.3\; \times \;10^{ - 9} } \right)\; \times \;Q^{3.5}$ When L = 40 m and Q = 300 m^3^/min, the diffusion distance of the DPM was the shortest. Therefore, the control effect of DPM was better under this air volume. At this point, the mathematical relationship between the diffusion distance C_40_ of CO gas and air volume Q is $$C_{40} = \left\{ \begin{gathered} 0.0012Q^{2} - 0.65Q\; + \;103.5\;(Q\; \le \;400) \hfill \\ 27.5\;{ + }\;\frac{1.5}{{1\;{ + }\;10\;(525 - Q)}}(Q\; > \;400) \hfill \\ \end{gathered} \right.$$ under the condition of L = 60 m and that the trolley is closer to the exit of the roadway. The control effect of DPM was better when Q = 200 m^3^/min, and thus CO gas could be discharged from the roadway as soon as possible. At this point, the mathematical relationship between the diffusion distance C60 of CO gas and air volume Q is $C_{60} = \;(3.14\; \times \;10^{ - 4} )\;Q^{2} - 0.2Q\; + \;51.5$.

### NO_x_ gas migration rule under long pumping and short pressure ventilation

**Figure 7:**
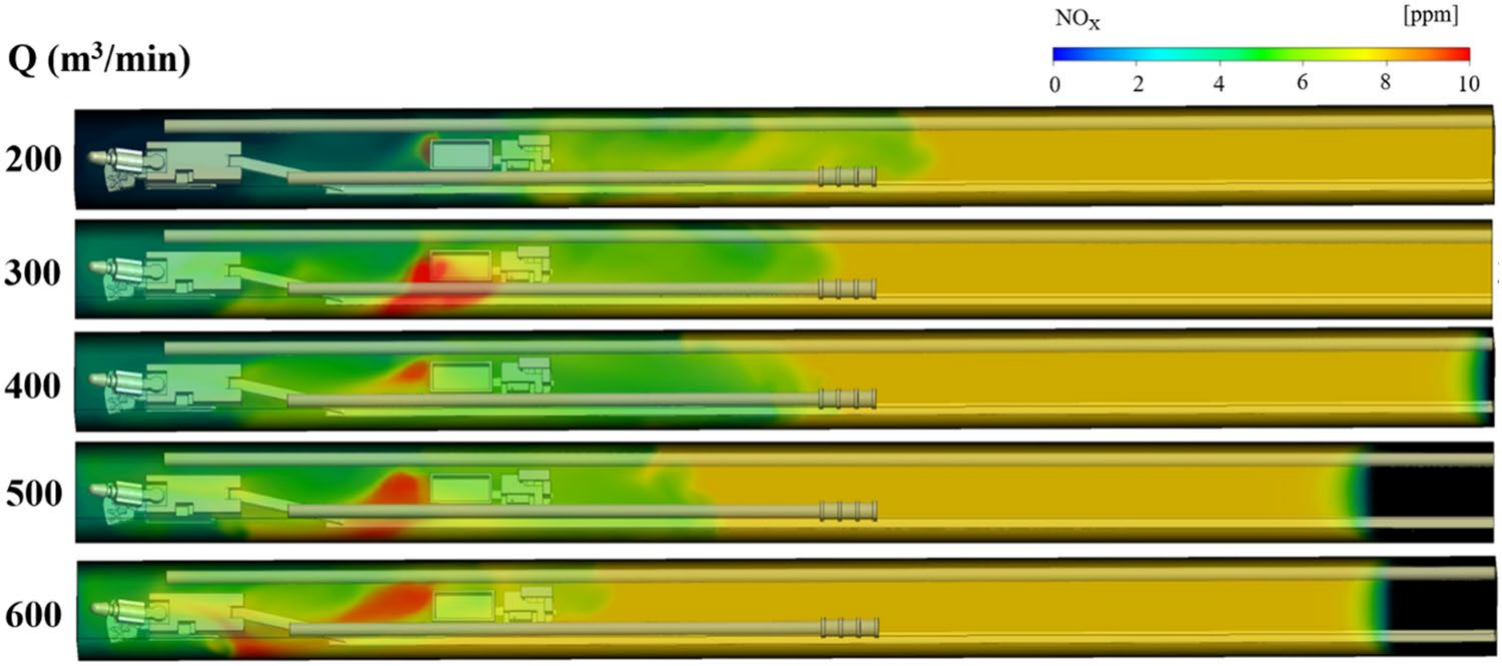
NO_x_ gas distribution under different air volumes Q at L = 20 m.

**Figure 8:**
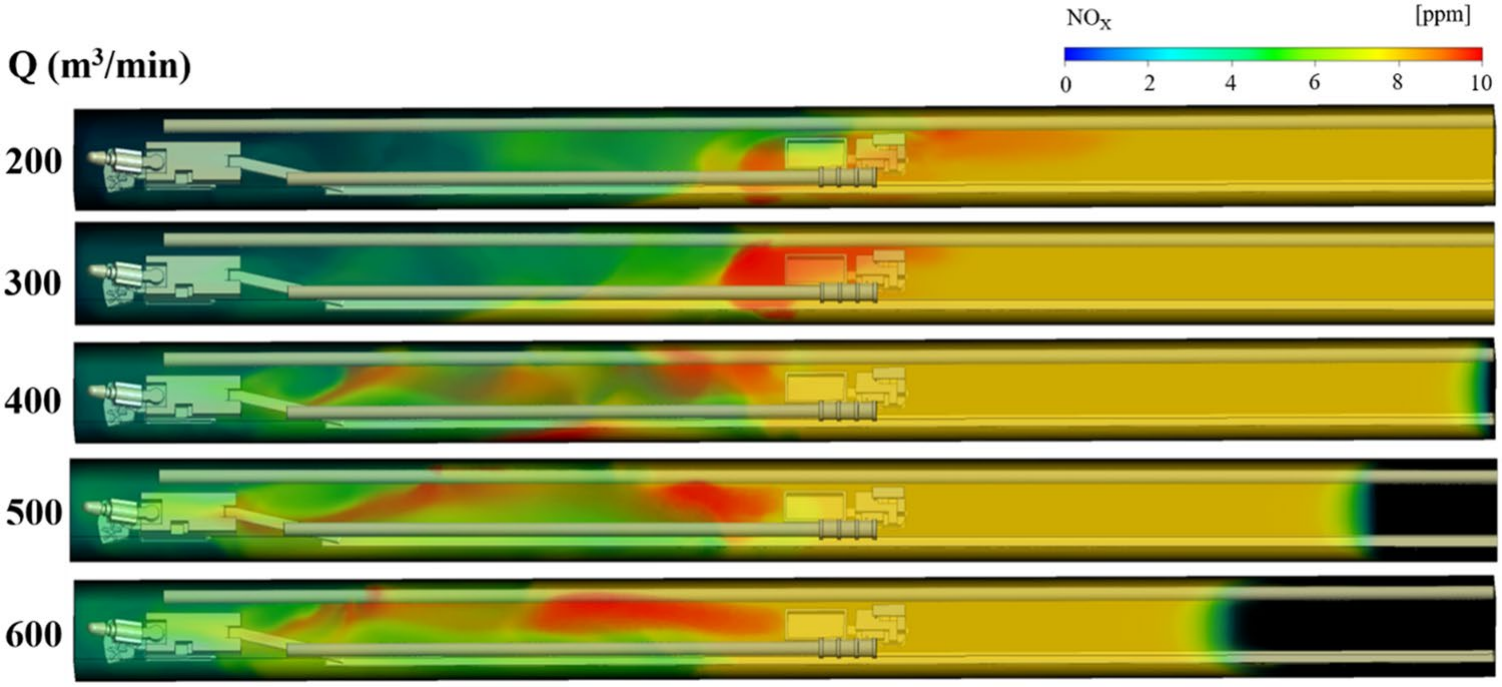
NO_x_ gas distribution under different air volumes Q at L = 40 m.

**Figure 9:**
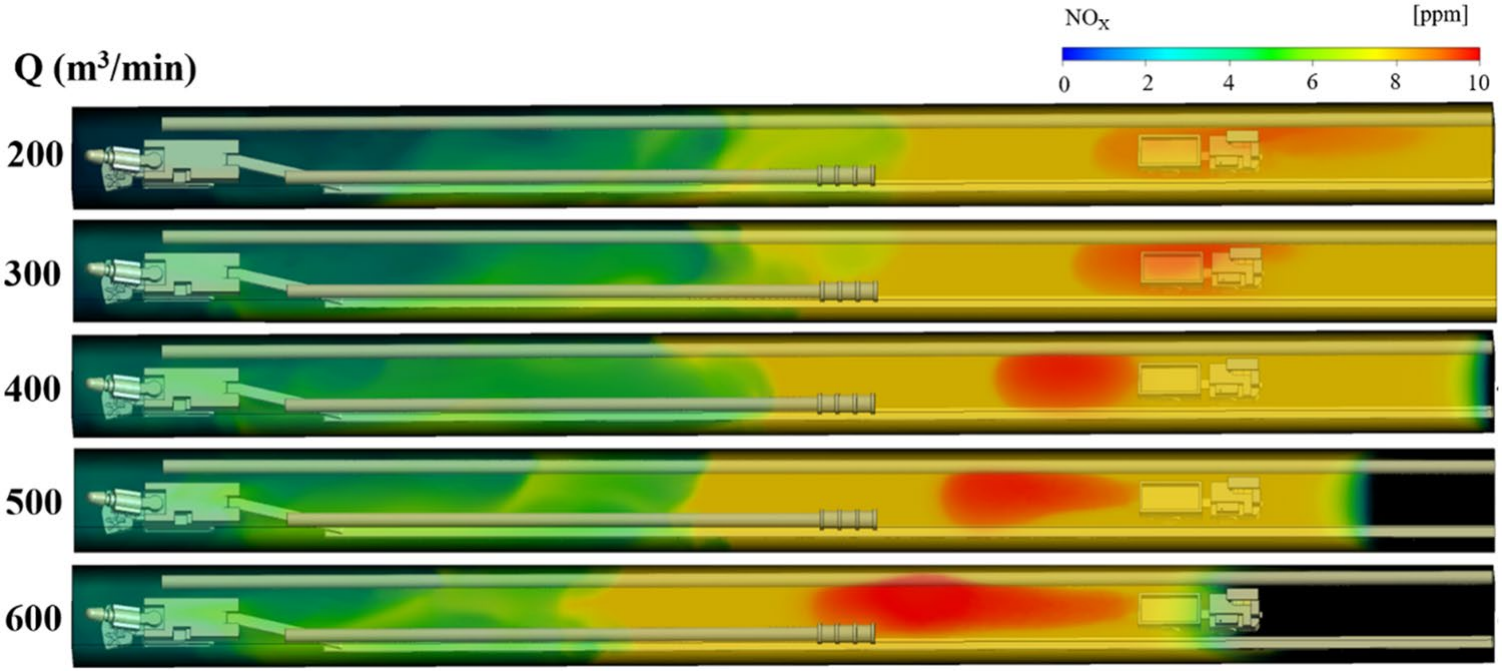
NO_x_ gas distribution under different air volumes Q at L = 60 m.

Under the condition of long suction and short pressure ventilation, the NO_X_ gas distribution law under different suction duct air volume Q and the distance L between the trackless rubber wheel car and headface, is shown in Figs. 7, 8 and 9. The colors of the different regions in the figures indicate the mass fraction of NO_x_ gas in different regions. To facilitate the study of the distribution of NO_x_ gas, the region where the NO_x_ gas mass fraction was greater than 8 ppm was called the high-volume-fraction NO_x_ region (red region in the figures); the region where the mass fraction was between 6 and 8 ppm was called the medium-volume-fraction NO_x_ region (yellow region in the figures), and the region where the mass fraction was less than 6 ppm was called the low-volume-fraction NO_x_ region (green region in the figures). The specific analysis is as follows:

1. When the distance L between the trackless rubber wheel car and the headface was constant, the distribution of NO_x_ and CO in the roadway were similar with the increase in the air volume Q of the extractive air duct. Taking L = 60 m as an example, when Q = 200 m^3^/min, because the pressure at the front end of the roadway was greater than that at the exit of the roadway, NO_x_ in the roadway migrated to the exit of the roadway as a whole with the airflow, and the diffusion distance of high-volume-fraction NO_x_ was 18.5 m. With the increase in Q, the pressure difference between the front end of the roadway and the exit of the roadway changed. When Q = 400 m^3^/min, owing to the negative pressure of the suction air duct, NO_x_ discharged by the trackless rubber wheel vehicle diffused toward the head direction, and the diffusion distance of the high-volume-fraction NO_x_ was 11.1 m. When Q = 600 m^3^/min, the pressure at the front end of the roadway was less than that at the outlet. Therefore, the NO_x_ discharged by the trackless rubber wheel vehicle diffused to the front end of the roadway, and the diffusion distance of the high-volume-fraction NO_x_ was 18 m. From Fig. 9, it can be inferred that when the ventilation mode in the roadway was dominated by pressure-in ventilation (Q < 400 m^3^/min), the volume fraction of NO_x_ gas was small, i.e., 8.8 ppm. When the ventilation mode in the roadway was dominated by extraction ventilation (Q > 400 m^3^/min), the volume fraction of NO_x_ gas was relatively large, i.e., 10 ppm.

(2) When the air volume Q of the extractive air duct was constant, the distance between the trackless rubber wheel car and the headface had varied effects on the distribution of NO_x_ gas with different mass fractions. With a change in L, the distribution of NO_x_ gas with low and medium volume fractions changed negligibly, but the distribution of NO_x_ gas with a high volume fraction changed significantly. From Figs. 7, 8 and 9, it can be inferred that NO_x_ gas with medium volume fraction was mainly distributed at the back end of the roadway, while the volume fraction of NO_x_ gas at the front end of the roadway was always relatively low because a part of NO_x_ gas was discharged from the roadway by the extractive air duct. The high-volume-fraction NO_x_ gas was mainly concentrated around the trackless rubber wheel vehicle. This was because the continuous emission of NO_x_ gas from the trackless rubber wheel vehicle was not discharged in time, resulting in an increased NO_x_ concentration with a volume fraction of 8 ppm.

(3) In summary, when L was constant, with the increase in air volume Q, the area with high-volume-fraction NO_x_ continued to expand and gradually approached the roadway head. At the same time, the low-volume-fraction NO_x_ region decreased with the increase in air volume Q. Therefore, when Q = 200 and 300 m^3^/min, the control effect of NO_X_ gas was better. By comparing the diffusion distance and volume fraction of low-, medium-, and high-volume-fraction NO_x_ gas for Q = 200 and 300 m^3^/min, it can be seen that when Q = 200 m^3^/min, the volume fraction of high-volume-fraction NO_x_ gas was slightly smaller, i.e., 8.5 ppm; in addition, the diffusion rate of high-volume-fraction NO_x_ gas was faster.

### DPM migration rule under long pumping and short pressure ventilation

**Figure 10:**
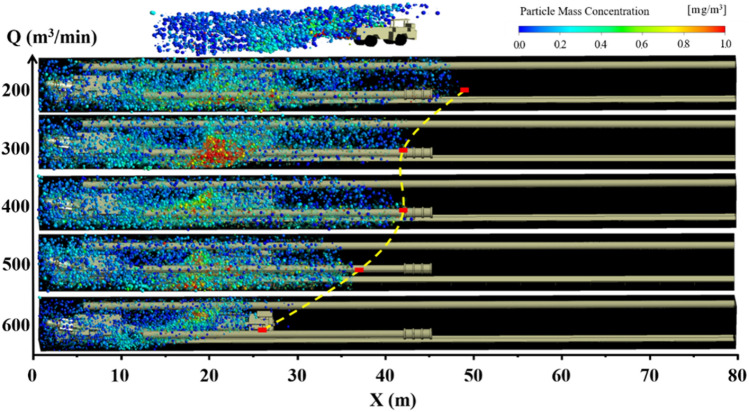
DPM distribution under different air volumes Q at L = 20 m.

**Figure 11:**
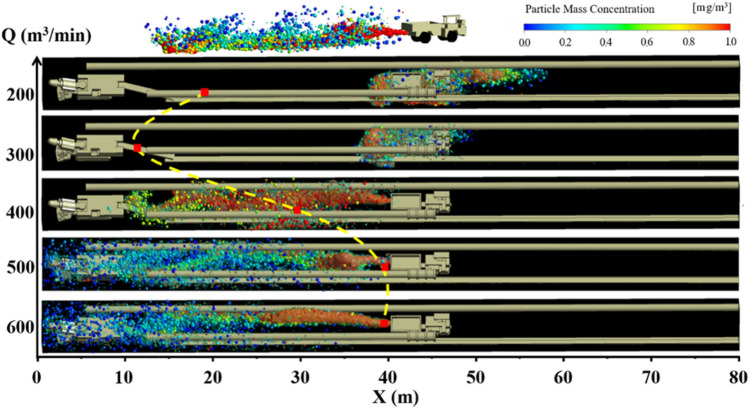
DPM distribution under different air volumes Q at L = 40 m.

**Figure 12:**
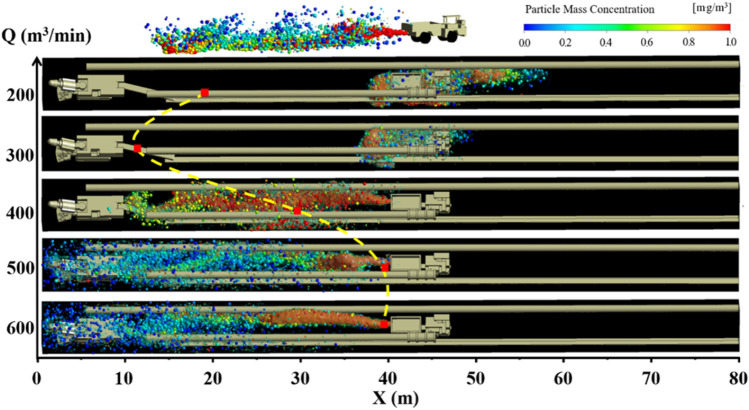
DPM distribution under different air volumes Q at L = 60 m.

Figures 10, 11 and 12 show the pollution evolution of DPM under long pumping and short pressure ventilation. The small balls in the figures are DPM. The colors represent the mass concentration of DPM, and the size is represented based on the legend in the upper left corner. Specific analysis is as follows:

1. When the distance L between the trackless rubber wheel car and the headface is constant, the diffusion distance and concentration of DPM in the roadway are related to L with an increase in the air volume Q of the extractive air duct. When L = 20 m, the diffusion distance of the DPM decreases with an increase in Q; this is because the DPM diffuses to the exit of the roadway when the ventilation mode in the roadway is primarily pressure-driven. When Q = 300 m^3^/min, the initial velocity of the DPM, after being discharged by the trackless rubber wheel vehicle, was close to and opposite to the velocity of the airflow. Therefore, the concentration of DPM was as high as 1800 ug /m^3^. When the ventilation mode in the roadway is dominated by the extraction type, as the air volume Q increases, the diffusion of DPM to the outlet of the roadway is blocked. In contrast, the increase in Q results in most of the DPM being discharged from the roadway by the extractive air duct; this reduces the concentration of DPM in the roadway. When L = 40 and 60 m, the diffusion distance of the DPM first decreased and then increased with an increase in Q.

(2) When the air volume Q of the exhaust duct is constant, the diffusion distance of DPM and the distance L between the trackless rubber wheel car and the headface are related. When Q ≤ 300 m^3^/min, with an increase in L, the diffusion distance of DPM first decreased and then remained unchanged. When Q > 300 m^3^/min, the diffusion distance of DPM decreased with an increase in L; this phenomenon is mainly related to the flow field at the location of the trackless rubber wheel car. When the trackless rubber wheel car was at the front end of the roadway, the wind energy in this area was large, and the trackless wheel car was closest to the outlet of the suction duct. Therefore, the diffusion distance of DPM was larger, and the concentration was smaller.

(3) In summary, when L = 20 m, DPM was diffused to the front end of the roadway owing to the negative pressure at the front end of the roadway, and the diffusion distance of the DPM decreased with an increase in Q. Therefore, when Q = 600 m^3^/min, the control effect of DPM was better. The mathematical relationship between the diffusion distance of DPM D_20_ and air volume Q is $D_{20} = - \;1.2e^{\frac{Q}{199}} + 50$. When L = 40 m, the diffusion distance of DPM first decreased and then increased with an increase in Q. When Q = 300 m^3^/min, the tail gas diffusion distance was the smallest, and the mathematical relationship between the diffusion distance D_40_ of DPM and air volume Q is $D_{40} = \left( { - 3 \times 10^{{{ - }6}} } \right)Q^{3} + 0.004Q^{2} - 1.32Q + 161$. When L = 60 m, the trolley was closer to the exit of the roadway. As the diffusion speed of DPM at the outlet of the roadway was the fastest when Q = 200 m^3^/min, DPM was discharged into the roadway as soon as possible; thus, the exhaust effect of DPM was the best when Q = 200 m^3^/min. The mathematical relationship between the diffusion distance of DPM D_60_ and the air volume Q is $D_{60} = \left( {2.36 \times 10^{{{ - }4}} } \right)Q^{2} - 0.193Q + 49$.

## Pollutant transport rule under long pumping and short pressure ventilation

**Figure 13:**
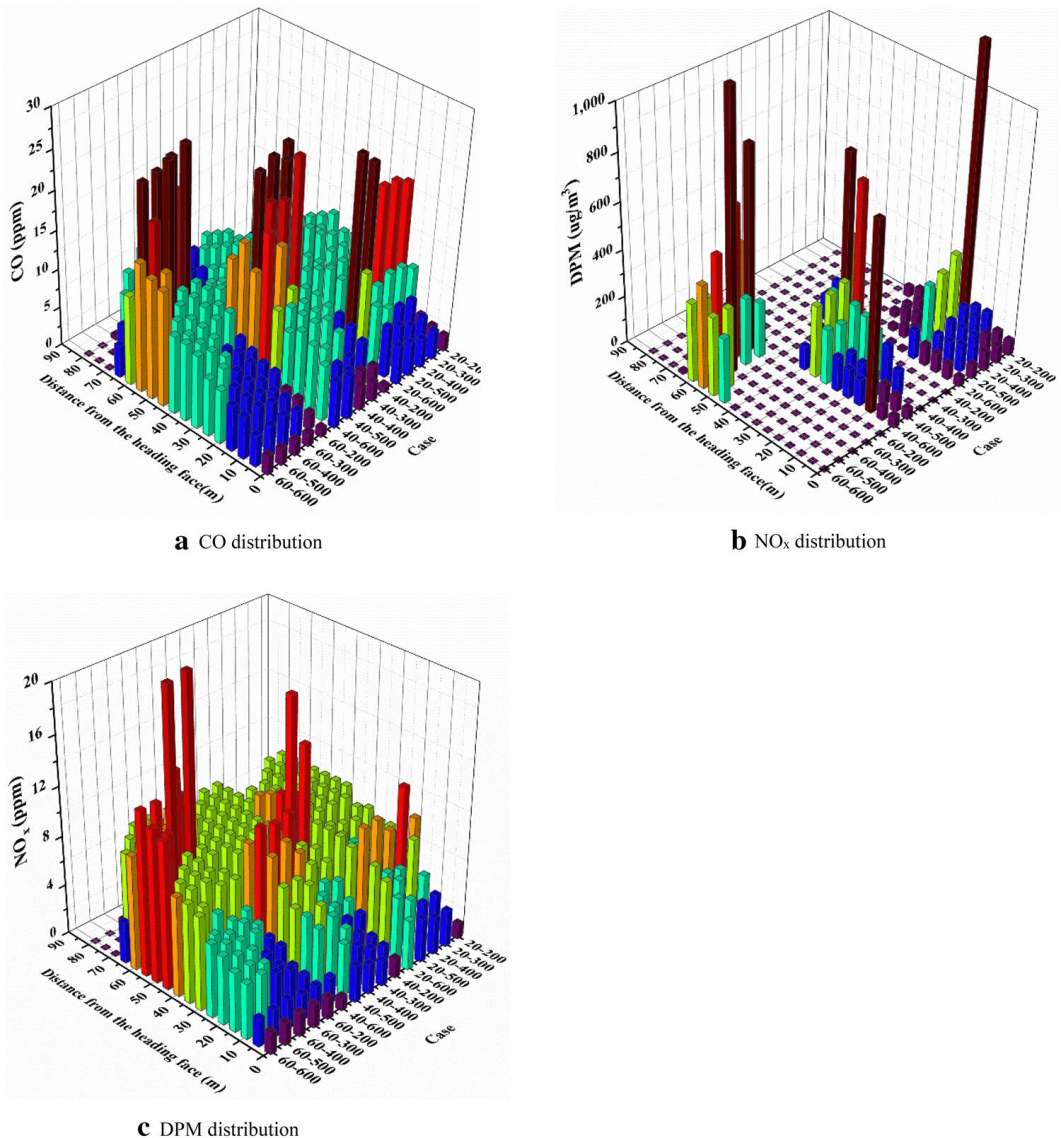
Variation of pollutant concentration along the path.

To explore the influence of air volume Q of different exhaust ducts and distance L between trackless rubber wheel cars and headfaces on pollutant diffusion under long suction and short pressure ventilation, we studied the diffusion laws of pollutants under different ventilation parameters. Figure 13 shows the variations in CO, NO_x_, and DPM concentrations along the path.

It can be inferred from Fig. 13 that:

1. Considering the diffusion range of pollutants, the diffusion range of DPM was smaller than that of CO, NO_x_, and other harmful gases. CO and NO_x_ gases were distributed throughout the roadway, while DPM was mainly distributed near the trackless rubber wheel vehicle; this is because the influence on DPM of gravity and other resistances was far greater than that on the gases, thereby hindering its diffusion in the roadway.

(2) As shown in Figs. 13a,b, the volume fractions of CO and NO_x_ in the area near the headface were less than 5 ppm. The volume fractions of CO and NO_x_ were related to the air volume Q and distance L between the trackless rubber wheel car and the headface. With an increase in the air volume Q, the volume fractions of CO and NO_x_ gradually increased. The volume fractions of CO and NO_x_ near the headface area gradually decreased with increasing distance L between the trackless rubber wheel vehicle and the headface. Except for the head-on area and the exhaust outlet area, the volume fractions of CO and NO_x_ were stable, and the volume fractions were 10 ppm and 8 ppm, respectively.

(3) As shown in Fig. 13c, DPM was mainly distributed around the exhaust outlet, and the concentrations exceeded the allowable concentration limit (0.1 mg/m^3^). When L = 20 and 40 m, the diffusion range of DPM was 40 m, which is much larger than the diffusion distance of DPM at L = 60 m. Owing to the slow diffusion rate of DPM, when the distance between the trackless rubber wheel car and the headface was 60 m and the air volume Q was 200 m^3^/min, DPM diffused to the exit of the roadway. At this point, the pollution of DPM at the working area of the driving face was small.

## Model verification

**Figure 14:**
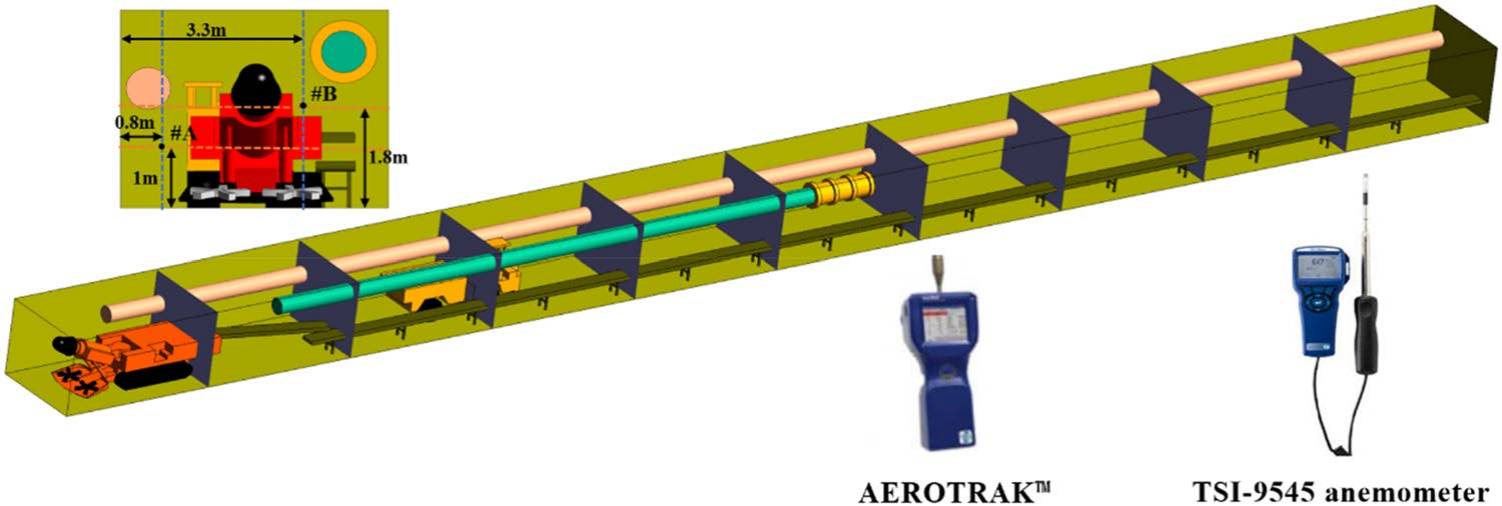
Layout of measuring points.

The TSI-9545 anemometer and AEROTRAK™9306 handheld laser particle counter were used to measure the wind speed in the roadway and the concentration of tail gas particles released by the trackless rubber truck. In order to avoid measurement error, each measuring point was sampled for three consecutive times, and the average value of the three measurements was taken as the final result. The layout of measuring points is shown in Fig. 14. The simulated and measured values of wind speed at the measuring points are shown in Table 2. The simulated and measured DPM values of the measured points are shown in Table 3. By comparing the measured wind speed and tail gas particles at each measuring point with the corresponding numerical simulation, it can be found that the relative error between the simulated value and the measured value is between 1.62 and 16.6%. The results show that the numerical simulation results are in good agreement with the field measurement results, and the numerical simulation results can effectively reflect the actual situation of the field.

**Table 2:** Simulated and measured values of airflow velocity at the measurement points.

Position of the measurement point	Velocity (m/s)	Distance between the measurement section and the heading face (m)									
		8	16	24	32	40	48	56	64	72	80
A (x, 0.8 m, 1.0 m)	Simulation results	3.04	0.52	0.82	0.11	0.24	0.39	0.23	0.21	0.23	0.22
	Measured results	3.09	0.54	0.84	0.12	0.22	0.41	0.25	0.23	0.24	0.24
	Relative error (%)	1.62	3.70	2.38	8.33	9.09	4.88	8.00	8.70	4.17	8.33
B (x, 3.3 m, 1.8 m)	Simulation results	0.33	1.13	0.31	0.22	0.05	0.14	0.22	0.22	0.21	0.21
	Measured results	0.32	1.15	0.33	0.24	0.06	0.15	0.24	0.23	0.20	0.20
	Relative error (%)	3.12	1.74	6.06	8.33	16.6	6.67	8.33	4.35	5.00	5.00

**Table 3:** Simulated and measured values of DPM at the measurement points.

Position of the measurement point	DPM concentration (ug /m^3^)	Distance between the measurement section and the heading face (m)									
		8	16 +	24	32	40	48	56	64	72	80
A (x, 0.8 m, 1.0 m)	Simulation results	45.6	98.1	245	60.8	35.6	23.1	6.3	3.4	3.1	2.1
	Measured results	46.9	100.1	240	63.3	37.3	24.1	6.8	3.8	3.5	2.2
	Relative error (%)	2.77	2.00	2.08	3.95	4.56	4.15	7.35	10.5	11.4	4.55
B(x, 3.3 m, 1.8 m)	Simulation results	32.2	102	214	179	35.6	28.2	10.6	6.7	5.2	3.4
	Measured results	34.8	110	224	183	39.6	30.1	12.2	6.9	5.8	3.6
	Relative error (%)	7.47	7.27	4.46	2.19	10.1	6.31	13.1	2.90	10.3	5.56

## Conclusion

1. When L = 20 m, the control effect of CO gas was optimum for each air volume Q. However, when Q = 600 m^3^/min, CO gas diffused rapidly to the front end of the roadway and was easily discharged from the driving face by the extractive air duct; therefore, the control effect of CO is better under this air volume. The diffusion distance of the DPM decreases with an increase in Q. When Q = 600 m^3^/min, the DPM diffused to the front end of the roadway owing to the negative pressure at the front end of the roadway and was discharged from the roadway through the suction air duct; therefore, the control effect of DPM is better under this air volume. With the increase in air volume Q, the high-volume-fraction NO_x_ region continued to expand, while the low-volume-fraction NO_x_ region continued to decrease. Therefore, when Q = 200 and 300 m^3^/min, the control effect of the NO_x_ gas is optimum. In summary, to comprehensively control and remove toxic and harmful substances in a comprehensive excavation face, the exhaust gas control effect in the roadway is optimum when Q = 600 m^3^/min.
2. When L = 40 m, the diffusion distance of CO gas first decreased and then increased with an increase in Q. When Q = 300 m^3^/min, the diffusion distance of the CO gas was the shortest; therefore, the control effect of CO gas under this air volume is better, and the diffusion law of NO_x_ gas is the same as that at L = 20 m. With the increase in air volume Q, the high-volume-fraction NO_x_ region continues to expand, while the low-volume-fraction NO_x_ region continues to decrease. Therefore, when Q = 200 and 300 m^3^/min, the control effect of the NO_x_ gas is good. The diffusion distance of DPM first decreases and then increases with an increase in Q. When Q = 300 m^3^/min, the exhaust gas diffusion distance was minimal. In conclusion, under the condition of L = 40 m, the exhaust gas control effect in the roadway is optimum when Q = 300 m^3^/min.
3. When L = 60 m, the trackless rubber wheel vehicle was closer to the exit of the roadway. When the ventilation mode in the roadway is mainly pressure-in ventilation, the exhaust gas is quickly discharged from the roadway, and the concentration of exhaust gas in the roadway reduces rapidly. When Q = 200 m^3^/min, the ventilation mode in the roadway was mainly pressure-in ventilation. At this air volume, CO gas and DPM moved to the outlet of the roadway, and the diffusion distance was small. In addition, compared with other air volumes, the high-volume-fraction NO_x_ region and medium-volume-fraction NO_x_ region were smaller under this air volume. Therefore, under the condition of L = 60 m, when Q = 200 m^3^/min, the exhaust gas control effect in the roadway is optimum.

## Data Availability

The datasets generated during and/or analysed during the current study are available from the corresponding author on reasonable request.

## List of symbols

$\rho$: Density of air (kg/m^3^)
$k$: Turbulent kinetic energy (m^2^/s^2^)
$\varepsilon$: Turbulent energy dissipation rate (m^2^/s^2^)
$\mu$: Laminar viscosity coefficient (Pa s)
$\mu_{t}$: Turbulence viscosity coefficient (Pa s)
$C_{1\varepsilon }$: Constants in the standard k-model (1.44)
$C_{2\varepsilon }$: Constants in the standard k-model (1.92)
$\sigma_{k}$: Constants in the standard k-model (1.00)
$\sigma_{\varepsilon }$: Constants in the standard k-model (1.30)
${\text{Sc}}_{t}$: Turbulent Schmidt number
$Y_{m}$: The mass fraction of the harmful gas
$$m_{p}$$: Particle mass
$\vec{u}$: Fluid phase velocity (m/s)
$$\vec{u}_{p}$$: Particle velocity (m/s)
$\rho_{{\text{p}}}$: Density of the particle (kg/m^3^)
$$\vec{F}$$: Additional force
$$d_{p}$$: Particle diameter (m)
$${\text{Re}}$$: Reynolds number
k: Turbulence kinetic energy (m^2^/s^2^)
ε: Turbulent dissipation rate (m^2^/s^2^)
$\tau_{r}$: Particle relaxation time
$u^{\prime }$: Random pulsation velocity (m/s)
$v^{\prime }$: Random pulsation velocity (m/s)
$w^{\prime }$: Random pulsation velocity (m/s)

## References

[1] Jiang W, Zhou G, Duan J, Liu D, Zhang Q, Tian F (2021). Synthesis and characterization of a multifunctional sustained release organic−inorganic hybrid microcapsule with self-healing and flame-retardancy properties. Appl. Mater. Interfaces.

[2] Wu W (2021). Coupled numerical model of hydraulic fracturing and seepage of soft coal based on elastoplastic damage. J. Shandong Univ. Sci. Technol. (Nat. Sci.).

[3] Liu R, Zhou G, Wang C, Jiang W, Wei X (2020). Preparation and performance characteristics of an environmentally-friendly agglomerant to improve the dry dust removal effect for filter material. J. Hazard. Mater..

[4] Ding J, Zhou G, Liu D, Jiang W, Wei Z, Dong X (2020). Synthesis and performance of a novel high-efficiency coal dust suppressant based on self-healing gel. Environ. Sci. Technol..

[5] Liu M, Lin M, Hu S, You X, Li L (2021). Effect of biomass surfactant on dehydration performance of low-rank coal and its mechanism. J. Shandong Univ. Sci. Technol. (Nat. Sci.).

[6] Liu L, Ma W, Wang W (2021). Dual damage mechanism of supercritical CO_2_ adsorption induced weakening effect on coal. J. Shandong Univ. Sci. Technol. (Nat. Sci.).

[7] Wang P, Tan X, Zhang L, Li Y, Liu R (2019). Influence of particle diameter on the wettability of coal dust and the dust suppression efficiency via spraying. Process Saf. Environ. Prot..

[8] Wang P, Gao R, Liu R, Yang F (2020). CFD-based optimization of the installation location of the wall-mounted air duct in a fully mechanized excavation face. Process Saf. Environ. Prot..

[9] Wang P, Han H, Liu R, Gao R, Wu G (2020). Effect of outlet diameter on atomization characteristics and dust reduction performance of X-swirl pressure nozzle. Process Saf. Environ. Prot..

[10] Gao RZ, Wang PF, Li YJ, Liu RH (2021). Determination of optimal blowing-to-suction flow ratio in mechanized excavation face with wall-mounted swirling ventilation using numerical simulations. Int J Coal Sci Technol..

[11] Liu R, Ji D, Zhou G, Liu Z, Xu Q, Ramakrishna S (2021). Electrospun nanofibers for personal protection in mines. Chem. Eng. J..

[12] Li Y, Wang P, Liu R, Gao R (2019). Optimization of structural parameters and installation position of the wall-mounted air cylinder in the fully mechanized excavation face based on CFD and orthogonal design. Process Saf. Environ. Prot..

[13] Chang P, Xu G (2017). A review of the health effects and exposure-responsible relationship of diesel particulate matter for underground mines. Int J Min Sci Technol..

[14] Li S, Zhou G, Liu Z, Wang N, Wei Z, Liu W (2020). Synthesis and performance characteristics of a new ecofriendly crust-dust suppressant extracted from waste paper for surface mines. J. Clean. Prod..

[15] Wang N, Wen Z, Liu M, Guo J (2016). Constructing an energy efficiency benchmarking system for coal production. Appl. Energy.

[16] Wang H, Cheng W, Sun B, Ma Y (2017). Effects of radial air flow quantity and location of an air curtain generator on dust pollution control at fully mechanized working face. Adv. Powder Technol..

[17] Ji C, He H, Ma H, Zhang Y, Zhao Y, Ma C (2004). Experimental study on the effect of fuel additives on diesel engine emissions. J. Beijing Univ. Technol..

[18] Lou D, Zhang Z, Tan P, Zhao Y, Zhang R (2010). Simulation study on regeneration balance of diesel particulate filter, Chinese internal combustion engine. Engineering.

[19] Kurnia JC, Sasmito AP, Wong WY, Mujumdar AS (2014). Prediction and innovative control strategies for oxygen and hazardous gases from diesel emission in underground mines. Sci. Total Environ..

[20] Zhang H, Fava L, Cai M, Vayenas N, Acuna E (2021). A hybrid methodology for investigating DPM concentration distribution in underground mines. Tunn. Undergr. Space Technol. Inc. Trenchless Technol. Res..

[21] Thiruvengadam M, Zheng Y, Tien JC (2016). DPM simulation in an underground entry: Comparison between particle and species models. Int. J. Min. Sci. Technol.

[22] Xu G, Chang P, Mullins B, Zhou F, Hu S (2018). Numerical study of diesel particulate matter distribution in an underground mine isolated zone. Powder Technol..

[23] Liu X, Nie W, Hua Y, Liu C, Guo L, Ma W (2021). Behavior of diesel particulate matter transport from subsidiary transportation vehicle in mine. Environ. Pollut..

[24] Chang P, Xu G, Zhou F, Mullins B, Abishek S, Chalmers D (2019). Minimizing DPM pollution in an underground mine by optimizing auxiliary ventilation systems using CFD. Tunn. Undergr. Space Technol..

[25] Liu C, Nie W, Liu X, Hua Y, Zhou W, Yu F, Niu W, Sun N, Xue Q (2021). Behavior of the particulate matter (PM) emitted by trackless rubber-tyred vehicle (TRTV) at an idle speed under different mov, ement conditions and ventilation optimization. Sci. Total Environ..

[26] Duan J, Zhou G, Yang Y, Jing B, Hu S (2021). CFD numerical simulation on diffusion and distribution of diesel exhaust particulates in coal mine heading face. Adv. Powder Technol..

[27] Zhang Q, Zhou G, Hu Y, Xing M, Zhang R, Wang P, Hu S (2021). Microwetting dynamic behavior and mechanism for coal dust based on low feld NMR method: A case study. Fuel.

[28] Zhou G, Zhang Q, Hu Y, Gao D, Wang S, Sun B (2020). Dust removal effect of negatively-pressured spraying collector for advancing support in fully mechanized coal mining face: Numerical simulation and engineering application. Tunn. Undergr. Space Technol..

[29] Han W, Zhou G, Wang J, Jiang W, Zhang Q, Kong Y, Miao Y (2021). Experimental investigation on combined modifcation for micro physicochemical characteristics of coal by compound reagents and liquid nitrogen freeze-thaw cycle. Fuel.

[30] Song SZ, Zhou G, Duan JJ, Zhang LC, Gao DH, Sun B (2021). Numerical simulation investigation on optimal dust-exhausting airflow volume in fully mechanized caving face of high-gas coal mine. Process Saf. Environ. Prot..

[31] Jiang H, Luo Y (2021). Development of a roof bolter drilling control process to reduce the generation of respirable dust. Int. J. Coal Sci. Technol..

[32] Gao R, Wang P, Li Y, Liu R (2021). Determination of optimal blowing-to-suction flow ratio in mechanized excavation face with wall-mounted swirling ventilation using numerical simulations. Int. J. Coal Sci. Technol..

[33] Sun Z, Chen L, Yu X, Liu G, Pan G, Li P, Ma H (2021). Study on optimization of shotcrete loading technology and the diffusion law of intermittent dust generation. J. Clean. Prod..

[34] Xie Z, Xiao Y, Jiang C, Ren Z, Li X, Yu K (2021). Numerical study on fine dust pollution characteristics under various ventilation time in metro tunnel after blasting. Build. Environ..

[35] Jing D, Jia X, Ge S, Zhang T, Ma M (2021). Numerical simulation and experimental study of vortex blowing suction dust control in a coal yard with multiple dust production points. Powder Technol..

[36] Ma Q, Nie W, Yang S, Xu C, Peng H, Liu Z, Guo C, Cai X (2021). Effect of spraying on coal dust diffusion in a coal mine based on a numerical simulation. Environ. Pollut..

